# Supplementary material for: Causal Relationship between Aging and Anorexia Nervosa: A White-Matter-Microstructure-Mediated Mendelian Randomization Analysis
Source: Biomedicines. 2024 Aug 16;12(8):1874. doi: 10.3390/biomedicines12081874 (PMC11351342; doi:10.3390/biomedicines12081874)
Supplement: Supplementary file 1 [file biomedicines-12-01874-s001.zip › Supplementary Figure/Figure S8/Leaveoneout plot of MR analysis for TL to ubm-b-1977.pdf]

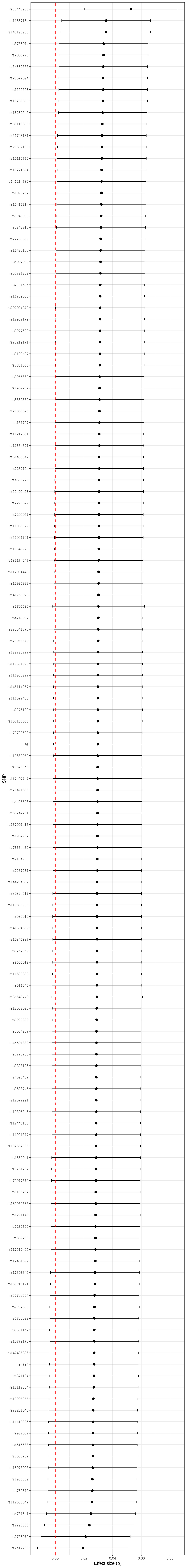
rs35446936
rs11557154
rs143190905
rs3785074
rs2056726
rs34550383
rs28577594
rs6669563
rs10768683
rs13230646
rs80116508
rs61748181
rs28502153
rs10112752
rs10774624
rs141214782
rs1023767
rs12412214
rs9940099
rs5742915
rs77732866
rs11426156
rs6007020
rs66731853
rs7221585
rs11769630
rs202034370
rs12932179
rs2977608
rs76219171
rs8102497
rs6881568
rs9955360
rs1907702
rs6659669
rs28363070
rs131797
rs11212631
rs11584821
rs61405042
rs2282764
rs4530278
rs59409453
rs2293579
rs7209057
rs11085072
rs56061761
rs10840270
rs185174247
rs117034449
rs12925933
rs41269079
rs7705526
rs4743037
rs376641875
rs76065543
rs139795227
rs112394943
rs111950327
rs145114957
rs111527438
rs2276182
rs150150565
rs73730598
A8
rs12369950
rs6590343
rs117407747
rs78491606
rs4498805
rs55747751
rs137901416
rs1957937
rs75664430
rs7164950
rs6587577
rs144204502
rs80324517
rs116863223
rs939916
rs41304832
rs10845387
rs3767952
rs9600019
rs11699829
rs611646
rs35640778
rs13062095
rs3093888
rs6054257
rs45604339
rs6776756
rs9398196
rs4695407
rs2538745
rs17677991
rs10805346
rs17445108
rs11991877
rs139669835
rs1332941
rs6751209
rs79977579
rs8105767
rs182059586
rs1291143
rs2230590
rs869785
rs117512405
rs12451892
rs17803849
rs188918174
rs56799554
rs2967355
rs6790988
rs3891167
rs10773176
rs142426306
rs4724
rs871134
rs11117354
rs10905255
rs77231040
rs11412296
rs932002
rs4616688
rs6536702
rs16978028
rs1985369
rs762679
rs117630647
rs4731541
rs7790856
rs2763979
rs9419958
SNP
0.00
0.02
0.04
0.06
0.08
Effect size (b)
